# Correlation between the setting of gating window width and setup accuracy in left breast cancer radiotherapy based on deep inspiration breath hold

**DOI:** 10.1002/acm2.14116

**Published:** 2023-08-04

**Authors:** Li Yucheng, Shao Yibo, Zhan Wenming, Jia Yongshi, Li Qiang, Qiu Lingyun, Huaxin Liu, YingHao Zhang, Ding jieni, Chen Weijun

**Affiliations:** ^1^ Cancer Center Department of Radiation Oncology Zhejiang Provincial People's Hospital (Affiliated People's Hospital) Hangzhou Medical College Hangzhou Zhejiang P. R. China; ^2^ Department of Cancer Radiotherapy and Chemotherapy Quzhou Hospital of Zhejiang Medical and Health Group (Zhejiang Quhua Hospital) Quzhou Zhejiang P. R. China

**Keywords:** cone beam computed tomography, deep inspiration breath‐hold, gated window width, left‐sided breast cancer, precision radiotherapy, setup error

## Abstract

Personalized precision irradiation of patients with left‐sided breast cancer is possible by examining the setup errors of 3‐ and 4‐mm gated window widths for those treated with deep inspiration breath‐hold (DIBH) treatment. An observational study was performed via a retrospective analysis of 250 cone‐beam computed tomography (CBCT) images of 60 left‐breast cancer patients who underwent whole‐breast radiotherapy with the DIBH technique between January 2021 and 2022 at our hospital. Among them, 30 patients had a gated window width of 3 mm, while the remaining 30 had a gated window width of 4 mm; both groups received radiotherapy using DIBH technology. All patients underwent CBCT scans once a week, and the setup errors in the left‐right (x‐axis), inferior‐superior (y‐axis), and anterior‐posterior (z‐axis) directions were recorded. The clinical‐to‐planning target volume (CTV‐PTV) margins of the two gating windows were calculated using established methods. The setup error in the Y direction was 1.69 ± 1.33 mm for the 3‐mm – wide gated window and 2.42 ± 3.02 mm for the 4‐mm – wide gated window. The two groups had statistically significant differences in the overall mean setup error (Dif 0.7, 95% CI 0.15–1.31, *t* = 2.48, *p*= 0.014). The Z‐direction setup error was 2.32 ± 2.12 mm for the 3‐mm – wide gated window and 3.15 ± 3.34 mm for the 4‐mm – wide gated window. The overall mean setup error was statistically significant between the two groups (Dif 0.8, 95% CI 0.13–1.53, *t*= 2.34, *p* = 0.020). There was no significant difference in the X‐direction setup error (*p* > 0.05). Therefore, the CTV‐PTV margin values for a 3‐mm gated window width in the X, Y, and Z directions are 5.51, 5.15, and 7.28 mm, respectively; those for a 4‐mm gated window width in the X, Y, and Z directions are 5.52, 8.16, and 10.21 mm, respectively. The setup errors of the 3‐mm – wide gating window are smaller than those of the 4‐mm – wide gating window in the three dimensions. Therefore, when the patient's respiratory gating window width is reduced, the margin values of CTV‐PTV can be reduced to increase the distance between the PTV and the organs at risk (OARs), which ensures adequate space for the dose to decrease, resulting in lower dose exposure to the OARs (heart, lungs, etc.), thus sparing the OARs from further damage. However, some patients with poor pulmonary function or unstable breathing amplitudes must be treated with a slightly larger gating window. Therefore, this study lays a theoretical basis for personalized precision radiotherapy, which can save time and reduce manpower in the delivery of clinical treatment to a certain extent. Another potential benefit of this work is to bring awareness to the potential implications of a slightly larger gating window during treatment without considering the resulting dosimetric impact.

## 1 INTRODUCTION

Breast cancer is one of the most common malignant tumors in women, posing a severe threat to their health.^1^, ^2^, ^3^ Adjuvant radiotherapy after surgery remains a standard therapeutic regimen for achieving locoregional control and improving overall survival in breast cancer patients.^4^, ^5^ However, this treatment method significantly increases the incidence of cardiovascular disease.^6^, ^7^, ^8^ The incidence of cardiovascular disease, the leading cause of death among breast cancer survivors, is greater in patients irradiated for left‐sided breast cancer than in those receiving irradiation for right‐sided breast cancer. Darby et al.^9^ analyzed the cause of death of more than 20 000 breast cancer patients, demonstrating that cardiovascular disease is the second leading cause of death in patients with a history of heart disease. Another study showed that the 10‐year cumulative death rate from cardiovascular disease is 16.9%, exceeding breast cancer‐related deaths (14.6%).^10^ Therefore, oncologists should focus on cardiac structure injury during radiation treatment planning for breast cancer. The application of deep inspiration breath hold (DIBH) technology in left breast cancer patients has gradually matured with the development of surface‐guided radiation therapy (SGRT) technology. Related studies have revealed that the DIBH technique of the SGRT system has certain advantages in reducing the incidence of cardiovascular disease in patients with left‐sided breast cancer.^11^, ^12^, ^13^, ^14^ However, few studies have focused on the width of the gating window and the accuracy of DIBH gating. This study primarily applies the Catalyst system, used for real‐time monitoring of breast cancer patients’ respiratory movements and control of radiation treatment, and studies the correlation between different gating window widths and setup errors under the DIBH, calculating the value of the margin from the clinical target volume (CTV) to the planning target volume (PTV), namely, the CTV‐PTV value. Thus, the relationships between setup error and gating window width can be obtained, which can greatly shorten the selection of gating window width in clinical practice and provide basic suggestions for the target margin with the same type of posture.

## 2 METHODS AND MATERIALS

### 2.1 Patients data

In this study, 250 cone‐beam computed tomography (CBCT) images from 60 left breast cancer patients treated in our hospital from May 2020 to June 2021 were enrolled and divided into two groups. Thirty patients were randomly selected using a random number generator of SPSS software (v20, SPSS Inc., Chicago, IL, USA) among the patients who underwent DIBH treatment with a 3‐mm gated window width at our institution, and the same method was implemented in the selection of 30 patients treated with a 4‐mm gated window width. All of the patients received the same type of breast radiotherapy, covering the whole breast, supraclavicular and infraclavicular regions, any part of the axillary bed at risk, and internal mammary nodes. The age of the 3‐mm gating window group was 47.80 ± 8.68 years, while that of the 4‐mm gating window group was 46.64 ± 7.12 years. The inclusion criteria included left‐sided breast cancer, no contraindications to radiotherapy, Karnofsky Performance Scale (KPS) score >70, age younger than 60 years, ability to completely understand the DIBH procedure, and ability to breath‐hold for more than 30 s. For all patients, simulated positioning and SGRT were completed with the Catalyst Systems v5.4.2 SP3 (C‐RAD Positioning AB, Uppsala, Sweden) with DIBH to reduce localization uncertainty during treatment delivery. The exclusion criteria included a breath‐holding time of fewer than 30 s, communication disorders, and other underlying diseases affecting radiotherapy.

### 2.2 Simulation positioning, target contour, and planning design

**FIGURE 1:**
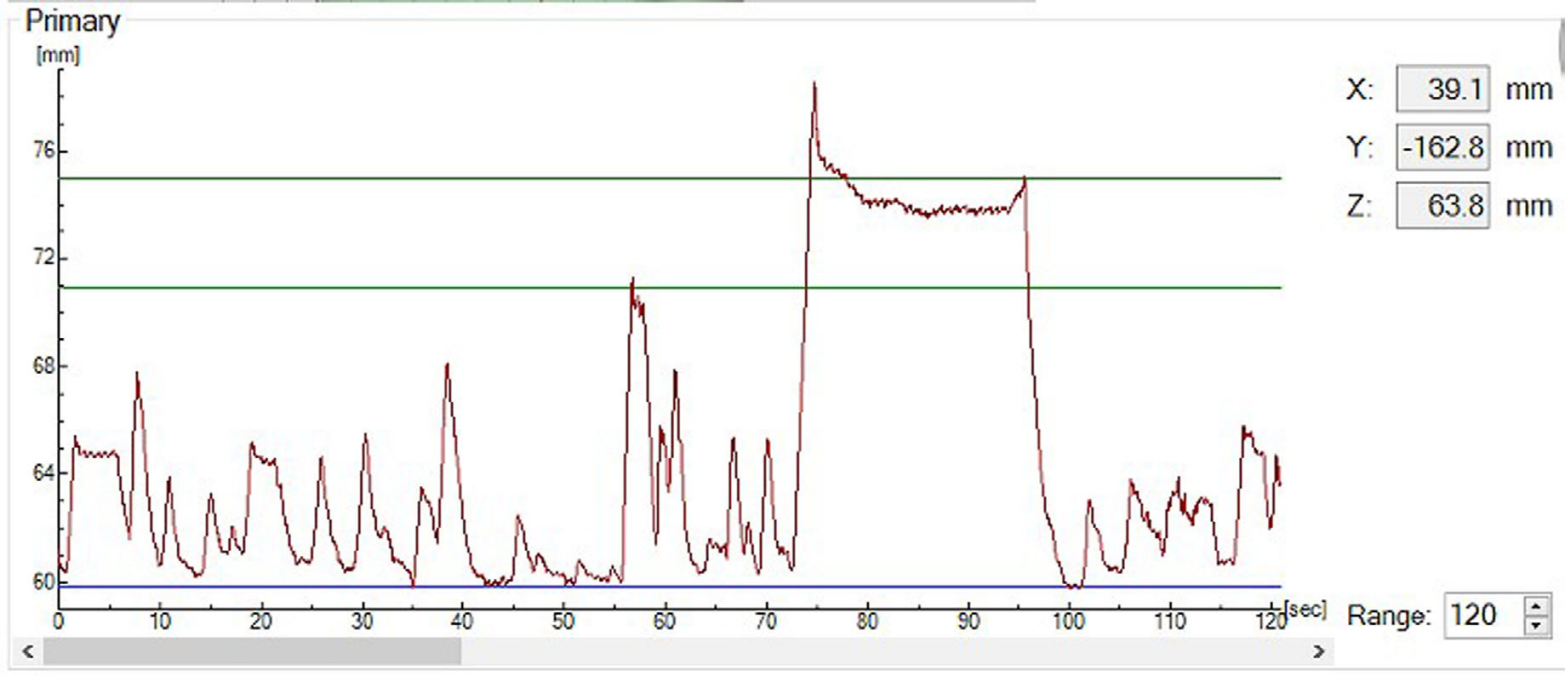
Gated window width baseline (blue line) and gated window width upper and lower thresholds (green line).

The patients were placed in a supine position on a vacuum cushion with an all‐in‐one board, with both arms fully abducted and externally extended. Patients underwent respiratory training prior to CT simulation to ensure regular breathing during the simulation and radiation treatment. The lower edge of the patient's xiphoid process was selected as the gate control point. Visual coaching via video goggles was provided to the patient to help with following the optimal DIBH breathing pattern in the CT simulator room, ensuring that the patient was trained until reproducible and stable deep inspiration was achieved. The baseline level and gating window width were defined according to the patient's ability to maintain a stable breath‐hold level and recorded in the patient database. According to the amplitude mode, the baseline of the gated window that triggers the CT scan on the workstation screen was set to approximately 80% (Figure 1). The selection of gating window width was based on the performance of the patient at the CT simulator. It is typically set to 3 mm during breathing training. However, some patients may find it challenging to meet this requirement during clinical practice. As a result, a gating window width of 4 mm is often used. During free breath (FB) CT acquisition, a virtual simulation procedure was carried out, and skin mark surrogates were generated. The prospective DIBH CT study was performed using a real‐time surface monitoring system (Sentinel v5.4.2 SP3, C‐RAD Positioning AB, Uppsala, Sweden) coupled with a large aperture CT simulator (version Discovery CT590, GE, Wisconsin USA). All CT scans were performed during end‐inspiratory breath‐hold. The CT acquisition can be accomplished within a single breath hold with modern CT simulator. Treatment planning CT scans were obtained at 5‐mm intervals from the ear to 2 cm below the diaphragm for each patient with the CT simulator. Despite the utilization of a 5 mm slice thickness during the CT scans, the 5 mm transverse CT scanning images were reconstructed into consecutive three‐dimensional images through software, generating reformatted sagittal and coronal CT images. The image data were then transmitted to the treatment planning system (TPS) for preplanning and design. The same principle was employed for the CBCT scans that were reconstructed into consecutive three‐dimensional images. The CT images were transferred to the MonacoV5.11 (Elekta AB, Stockholm, Sweden) TPS in DICOM format. In this study, physician automatically delineated the OARs using an AccuContour (version 3.2, Manufactured by MANTEIA, XiaMen, China) automatic software sketching, and the target was delineated under the National Research Council of Canada's Division of Radiation Oncology (NRC Oncology) and the International Commission Radiological Units (ICRU).^15^, ^16^ All of the CTV were combined into one target named as CTVs, and expanded uniformly by 5 mm in all directions and then retracted 5 mm subcutaneously to produce the PTV. The prescribed doses delivered to the tumor were 50 Gy/25 f. The plans were executed through the utilization of volumetric modulated arc therapy (VMAT), whereby they were formulated with a 6‐MV photon beam that was normalized to encompass 95% of the PTV with the prescribed dose. The same senior medical physicist first designed the plan, and then the radiation oncologist and the medical physicist jointly evaluated the plan before implementation to ensure plan quality. After plan approval, it was sent to the linear accelerator (Infinity, Elekta AB, Stockholm, Sweden) for planning verification and treatment.

### 2.3 Setup, CBCT workflow, and data collection

**FIGURE 2:**
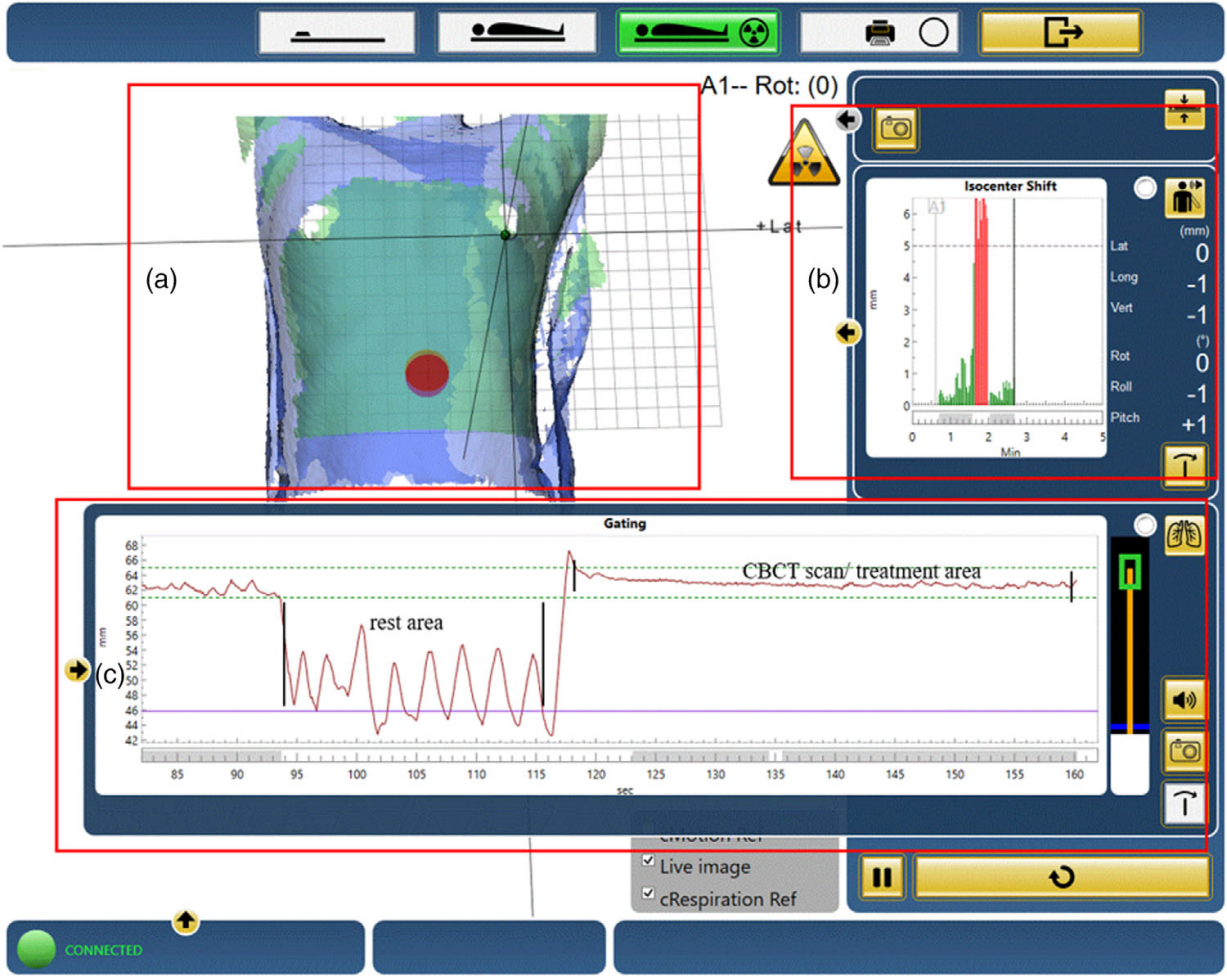
Illustration of the patient treatment using the C‐RAD. (a) shows the patient contour information: the blue contour is the live image, the green contour is the reference image, and the red point on the patient's surface is the gating window point. (b) shows the isocenter shift: the red setup error information indicates periods of rest between breath holds, and the green setup error information indicates a CBCT scan or radiation treatment. (c) shows the patient's breath curve during radiation treatment.

Patients were kept in the same supine position as during the CT simulator for the treatment. The laser was aligned to the marks placed on the patient's skin in the FB state and verified by the Catalyst system at the Linear‐accelerator end. The Catalyst system is capable of monitoring motion in six dimensions, the patient setup criteria were as follows: the deviation of translation ≤5 mm, and the deviation of rotation ≤3°. If the predetermined deviation thresholds were surpassed, the radiation technologist proceeded to reposition the patient until the deviation was within the acceptable range of error. Once the positioning was deemed satisfactory, the radiation technologist concluded the procedure and exited the treatment room. CBCT scans were performed with a “chest M20 fast” protocol and an M20 collimator, which yielded an axial field of view (FOV) of 41 cm with a length of 26 cm. The patient maintained a stable and reproducible breath curve within the set gating window and started the CBCT scan manually (as shown in Figure 2). During the entire scan, the operator had to ensure that the patient's breath curve was within the gating window; otherwise, the scan needed to be manually paused, and the above actions repeated until the entire CBCT scan was completed. The patient typically needs 2 to 3 breath‐hold cycles to complete a CBCT scan. The setup error data were obtained from the automated fusion of the CBCT and planning CT with grayscale registration, which was then manually fine‐tuned and aligned with the PTV by the senior physician. The region of interest for this automatic image registration included the entire PTV, left lung, and sternum. The patient's setup errors along the x‐axis (left‐right), y‐axis (inferior‐superior), and z‐axis (anterior‐posterior) were recorded for gated window widths of 3 and 4 mm and determined at the treatment console. Each patient underwent a weekly CBCT scan before radiation therapy. All patients included in the study underwent approximately the same number of CBCT scans. CBCT was used to verify and calibrate the setup under DIBH, and SGRT involved digital visualization of the patient's surface for setup as well as tracking intrafraction motion. All patient treatments with DIBH were triggered by the Catalyst “Response” gating interface within a preset gating window for Liner‐accelerator beam‐on therapy, as illustrated in Figure 2. The Catalyst system was used for real‐time monitoring of the patient's respiratory movements and control of the radiation treatment, which can significantly reduce the breathing amplitude and keep the tumor in a relatively quiescent state.

### 2.4 Statistical analysis

The reported setup error was derived from all data, which was subjected to statistical analysis using SPSS software. Data with a normal distribution and homogeneity of variance were analyzed by the independent sample t test between two groups and are expressed as $\bar{x}$ ± s. A *p‐*value < 0.05 was considered to indicate statistical significance. Prism software (GraphPad Prism 5, GraphPad Software, Inc.) was used for graphing the data.

## 3 RESULTS

### 3.1 Comparison of setup errors with 3‐ and 4‐mm gating window

**FIGURE 3:**
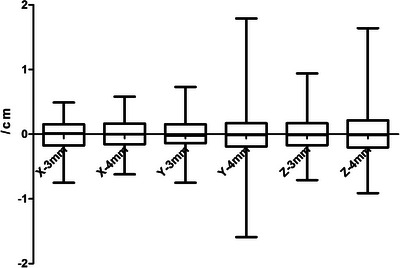
The distribution of setup errors in the X, Y, and Z direction.

The setup error in the Y direction with 3‐ and 4‐mm gating window widths was 1.69 ± 1.33 mm and 2.42 ± 3.02 mm, respectively, and the difference was statistically significant (Dif 0.7, 95% CI 0.15–1.31, *t* = 2.48, *p* = 0.014). In the Z direction, the setup error values were 2.32 ± 2.12 mm for the 3‐mm gating window width and 3.15 ± 3.34 mm for the 4‐mm gating window width, with a significant difference between the overall means of the two groups (Dif 0.8, 95% CI 0.13–1.53, *t*= 2.34, *p* = 0.020). The difference between the two groups in the X‐direction was not significantly different (*p* > 0.05). Table 1 presents the detailed values for the two groups, and Figure 3 illustrates the distribution of the means of the two groups.

**TABLE 1:** Comparison of setup error values in the three‐dimensional direction between the two groups (mm).

	3 mm	4 mm	Dif 95%CI	*t*	*p*
X (mm)	1.82 ± 1.35	1.82 ± 1.38	0 (−0.3 to 0.3)	0.03	0.978
Y (mm)	1.69 ± 1.33	2.42 ± 3.02	0.7 (0.15–1.31)	2.48	0.014
Z (mm)	2.32 ± 2.12	3.15 ± 3.34	0.8 (0.13–1.53)	2.34	0.020

### 3.2 Comparison of the margin value of the target with 3‐ and 4‐mm gating window

According to Van Herk et al.'s study on radiotherapy setup errors and target margins, random errors affect the distribution of the target dose, while systematic errors affect the accumulation of the target dose.^17^ Subsequently, they derived a margin formula to guarantee that 90% of patients in the population received a minimum cumulative CTV dose of at least 95% of the prescribed dose. Specifically, the margin value by which the CTV was expanded to form the PTV should be$\;M_{\mathrm{PTV}}=2.5\Sigma +0.7\sigma$, Σ represents the population systemic error, the standard deviation of the individual systematic error, and σ represents the population random error, the standard deviation of the individual random error. According to this formula, we deduced the margin values of the target areas for the 3‐ and 4‐mm gated windows (Table 2).

**TABLE 2:** The margin value of CTV‐PTV in three directions between two group.

	group	Σ	σ	$M_{PTV}$
X	3 mm	1.82	1.35	5.51
	4 mm	1.82	1.38	5.52
Y	3 mm	1.69	1.33	5.15
	4 mm	2.42	3.02	8.16
Z	3 mm	2.32	2.12	7.28
	4 mm	3.15	3.34	10.21

## 4 DISCUSSION

We have entered the era of image‐guided high‐precision radiotherapy in tumor radiotherapy with the rapid development of medical imaging and computer technology. Commonly used image‐guided radiotherapy devices include CBCT systems and electronic portal imaging (EPID); however, both types of scans will expose the patient to extra radiation doses, and neither is able to monitor tumors in real‐time. According to large‐scale experimental tests, the SGRT technique does not impose additional radiation and is advantageous for guiding the radiotherapy setup, monitoring inter‐ and intrafraction target movements in real‐time, and reducing the scanning frequency of CBCT in conjunction with respiratory gating techniques.^10^ This study analyzed the setup errors for 3‐ and 4‐mm gating window widths with the DIBH technique. The results indicate that the setup error of the 3‐mm gated window width in all three dimensions is smaller than that of the 4‐mm gated window width, implying that when the width of the patient's gating window decreases, the CTV‐PTV margin value can be reduced.

Table 1 and Figure 3 reveal that the differences in the setup error values between the 3‐ and 4‐mm gated window widths are statistically significant in the Y and Z directions but not in the X direction. This suggests that the choice of 3‐ or 4‐mm gating window width for left breast cancer radiotherapy has little effect on the patient's setup error values in the left‐right directions, while the inferior‐superior and anterior‐posterior directions have some impact. In this study, the 4‐mm gating window width allowed the patient greater freedom in locating the breath‐holding equilibrium point, resulting in a scattering of the range of motion of their target area due to respiratory motion. The relevant literature indicates that the respiratory movement of patients with thoracic tumors may vary significantly in the Y and Z directions as the chest rises and falls, while there is little effect in the X direction.^18^, ^19^ This demonstrates that decreasing the width of the gating window can minimize set‐up errors in the Y and Z directions, but the reduction in the X direction is not noticeable. There are currently numerous formulas for calculating the CTV‐PTV margin; among them, Van Herk^17^ considered the systematic error and random error in treating patients and deduced a margin formula of $M_{\mathrm{PTV}}=2.5\Sigma +0.7\sigma$. This formula guarantees that 99% of the CTV will receive 95% of the prescription dose. Numerous researchers have calculated the CTV‐PTV margin based on this formula,^18^, ^19^, ^20^, ^21^, ^22^ which produces CTV‐PTV margins in the X, Y, and Z directions of 5.51, 5.15, and 7.28 mm, respectively, for a 3‐mm – wide gating window and 5.52, 8.16, and 10.21 mm, respectively, for a 4‐mm – wide gating window. Reducing the width of the gating window directly leads to a reduction in the target area's margin, ensuring a sufficient dose to the target area while lowering the dose to the OARs. Additionally, Table 2 shows that the random setup error for a 3‐mm gated window is less than that of a 4‐mm gated window, indicating that reducing the gated window width can further reduce the setup error fluctuation range.

This study identifies significant differences in the Y and Z directions between the setup error values of the 3‐ and 4‐mm gated window widths via three‐dimensional analysis. Therefore, in cases where the same body position fixation method is used, when the width of the patient's respiratory gating window is reduced, the CTV‐PTV margin value can be reduced to a certain extent to further reduce the damage to the OARs. Some patients undergoing radiotherapy for breast cancer may have poor respiratory amplitude stability due to age or other reasons; therefore, this study recommends the three‐dimensional CTV‐PTV margin values for the 4‐mm gate window width. We can use the recommended value of the CTV‐PTV margin in the three dimensions with a 3‐mm gating window width on, for example, young patients with stable respiratory amplitude. In addition to relying on special equipment, the patient's cooperation, particularly her breath‐holding time, stability, and ability to perform a deep inhalation, may greatly affect the treatment curative effect and side effects during the implementation of DIBH technology.^22^, ^23^, ^24^, ^25^ It is believed that only patients who can hold their breath for more than 30 s after training can implement this technique to obtain sufficient breath‐holding time in clinical practice. An appropriate gating window width, such as 3 mm or 4 mm, is usually set. Increasing the width of the gating window increases the motility of the target, amplifies the interplay effect, and increases the gradient index effect, but it is worth increasing the width of the gating window and sacrificing some dosimetric accuracy to achieve the required breath‐hold time and stability for DIBH treatment. Nevertheless, this study only examined the setup errors for gating window widths of 3 and 4 mm, not for 2 and 5 mm. Further research into the setup errors for 2 and 5 mm gating window widths would help us provide more evidence for personalized precision radiotherapy.

## 5 CONCLUSION

This study revealed that the setup error of the 3 mm gating window in three dimensions is smaller than that of the 4 mm gating window. Therefore, when the patient's respiratory gating window width is reduced, the CTV‐PTV margins can be reduced to increase the distance between the PTV and the OARs, ensuring adequate space for the dose to decrease, resulting in lower dose exposure to the OARs (heart, lungs, etc.) and thus sparing the OARs from further damage. However, certain patients, such as those with poor pulmonary function or an unstable breathing amplitude, must be treated with a slightly larger gating window. Therefore, this study lays a theoretical basis for personalized precision radiotherapy that can save time and manpower in the delivery of clinical treatment to a certain extent.

## AUTHOR CONTRIBUTIONS

Yucheng Li: Manuscript drafting, editing, and statistical analysis. Weijun Chen: Design, supervision, data interpretation, and critical review. Yongshi Jia, Hanchu Xiong, Shao Yibo, Wenming Zhan, and Lingyun Qiu: Patient surveillance and data acquisition. Qiang Li, Huaxin Liu, YingHao Zhang, and Ding Jieni: Literature search. All authors read and approved the final manuscript.

## CONFLICT OF INTEREST STATEMENT

The authors declare no conflicts of interest.

## ETHICS STATEMENT

This study was approved by the ethics institutional review board of Zhejiang provincial people's Hospital and conducted per the ethical standards of the Declaration of Helsinki.
